# Sexual health in menopausal women with symptoms of pelvic floor disorders

**DOI:** 10.18332/ejm/194171

**Published:** 2024-10-29

**Authors:** Rocío Adriana Peinado Molina, Sergio Martínez Vázquez, Antonio Hernández Martínez, Juan Miguel Martínez Galiano

**Affiliations:** 1 Department of Nursing, University of Jaen, Jaen, Spain; 2 Department of Nursing, Physiotherapy and Occupational Therapy, Faculty of Nursing, University of Castilla-La Mancha, Ciudad Real, Spain; 3 Consortium for Biomedical Research in Epidemiology and Public Health (CIBERESP), Madrid, Spain

**Keywords:** sexual health, menopause, pelvic floor disorders, sexual dysfunction

## Abstract

**INTRODUCTION:**

Sexual dysfunction in women is usually associated with the menopausal transition and menopause; however, there are factors that can also influence the sexual function of women in menopause. The aim of this study is to determine the association between pelvic floor disorders and sexual dysfunction in women in menopause.

**METHODS:**

A cross-sectional study was carried out in Spain with menopausal women recruited by convenience sampling. Data were collected on background and health status. To evaluate the presence of pelvic floor problems, the Pelvic Floor Distress Inventory (PFDI-20) was used. Regarding the evaluation of female sexual function, the validated Sexual Function of Women (FSM-2) tool was used. Crude (OR) and adjusted odds ratios (AOR) were obtained using the SPSS 28.0 statistical program.

**RESULTS:**

A total of 197 women participated. The mean age was 57.7 years (SD=8.4), 51.3% (101 women) reported experiencing some form of sexual dysfunction. Despite this, the majority (79.5%; 155 women) indicated that they were satisfied with their sexual health. However, 25.5% (50 women) mentioned they faced difficulties when trying to initiate sexual intercourse. Additionally, 22.9% (45 women) reported having moderate to severe issues achieving orgasm. Furthermore, 29% (57 women) stated that they had never or only occasionally felt arousal in the past month. Women who experienced urinary incontinence and pelvic pain had a higher frequency of sexual dysfunction. The main associated factor observed was the risk of pelvic floor dysfunction through the PFDI-20 scale. For each point of this instrument, there was a small but increased risk of sexual dysfunction (OR=1.01; p<0.001). Type of birth or maternal disorders, such mental illness or gastrointestinal disorder, did not show any statistical association with sexual dysfunction.

**CONCLUSIONS:**

Pelvic floor dysfunctions symptoms in menopausal women are associated with their sexual health. Pelvic floor dysfunctions that influence sexual function are colorectal, urinary, and prolapse. Pelvic floor disorders such as urinary incontinence and pelvic pain are those that most influence sexual function.

## INTRODUCTION

Sexuality, according to the World Health Organization (WHO), can influence a woman’s quality of life by having effects on her emotional, physical, and psychological well-being, one of its fundamental components^1^. There is a widespread and mistaken belief that as people age, they lose both interest in and ability to have sexual relations^2^. However, many women continue to maintain sexual interest; in fact, 76% of women aged 42–52 years consider sex to be important to them^3^. Thus, approximately 67% of American women aged 65–74 years express the importance they give to sex^4^. At the same time, it is widely recognized that sexual activity declines during the transition to menopause and beyond^4-7^. Despite the importance of sexual function in women at this stage of life, sexual dysfunction also tends to increase with age^8^. Sexual dysfunction in women is usually associated with the menopausal transition and menopause since they are times characterized by hormonal, physiological, and social changes^8^. Up to 85% of women experience symptoms linked to this transition that vary in intensity and can affect their quality of life^9^. From a physiological point of view, the variation in hormonal levels negatively affects the elasticity of the vaginal mucosa and vaginal lubrication, which leads to vaginal atrophy. Additionally, as we age, the likelihood of weakening of the pelvic floor increases, leading to conditions such as dyspareunia, chronic pelvic pain, and pelvic organ prolapse^8^.

Pelvic floor problems encompass various problems that can affect this muscular structure. These disorders include situations such as urinary incontinence, pelvic organ prolapse in women, fecal incontinence, and pelvic-perineal regional pain syndrome, among others^10^. It is a public health problem with a high prevalence and can affect a quarter of adult women^11^ negatively, influencing the sexual function of women with these symptoms^8,12,13^.

Women with pelvic floor dysfunction may experience a decrease in their quality of life and compromised sexual function due to factors such as a negative perception of their body image, anxiety related to incontinence during sexual activity, feelings of shame, dyspareunia, and difficulties in achieving a satisfying orgasm^14-16^.

Since the relationship between pelvic floor disorders and sexual function in women during menopause has been investigated to a limited extent^12,13^, and considering that there are contradictory findings, and further research on the topic is recommended^17,18^. The aim of the present study was to investigate the association between pelvic floor disorders and sexual dysfunction in women during menopause.

## METHODS

### Study design, setting and participants

A cross-sectional study was carried out in Spain in 2022 with women who were in menopause. Women with difficulties in the Spanish language or with mental and cognitive problems that affected data collection for the study, were excluded. The women were recruited by convenience sampling.

To carry out this research, it was necessary to recruit at least 196 women based on the following criteria: a confidence level of 95%, an absolute margin of error of 7%, a prevalence in the population of menopausal women of 50% (this criterion is used because it is the most conservative since no recent prevalence data have been located and in addition, different dysfunctions with different prevalence are addressed) in relation to sexual dysfunction.

### Data sources, measurement and variables

The recruitment of women was carried out in places frequented by them, such as health centers or GP surgeries, women’s associations and information centers, and after their interest in participating in the research, informed consent was obtained, followed by interviews carried out by two trained observers using a questionnaire as a script. The selection of women was carried out consecutively.

Data on sociodemographic and work characteristics, background and health status, as well as habits and lifestyle, were collected through a specifically designed and previously pilot-tested questionnaire, which did not require any posterior modification to launch the data collection.

Additionally, to evaluate the presence of pelvic floor problems, the Pelvic Floor Distress Inventory (PFDI-20) was used, which consists of 20 items composed by 3 subscales. The UDI-6 subscale, composed of 6 items, evaluates urinary symptoms, the CRADI-8 subscale evaluates colorectal symptoms, while the POPDI-6 subscale, with another 6 items, evaluates prolapse symptoms, all subscales have one score maximum of 100 points each. The PFDI-20 has a total score of 300 points, and a higher score indicates a greater symptom burden^19^. To determine the presence of prolapse, an affirmative response to question 3 (‘Usually have a bulge or something falling out that you can see or feel in your vaginal area?’) of the PFDI-20 Spanish version was considered. Fecal incontinence was assessed by PFDI-20 responses 9 (‘Usually lose stool beyond your control if your stool is well formed?’) or 10 (‘Usually lose stool beyond your control if your stool is loose?’), urinary incontinence by PFDI-20 responses 16 (‘Usually experience urine leakage associated with a feeling of urgency, that is, a strong sensation of needing to go to the bathroom?’), 17 (‘Usually experience urine leakage related to coughing, sneezing, or laughing?’), or 18 (‘Usually experience small amounts of urine leakage (that is, drops)?’), and pelvic pain by PFDI-20 responses 20.

Regarding the evaluation of female sexual function, validated in Spanish women, the Women’s Sexual Function tool (FSM-2) was used. This self-administered questionnaire consists of 14 questions. Responses were rated on a Likert scale from 1 to 5 and integrated into domains. The questions included in the sexual activity evaluation domains have a score from 1 to 5, while those in the descriptive domain do not have a quantitative value and serve to recognize important aspects, such as sexual frequency or the existence of a partner, as well as questions for the diagnosis of sexual dysfunctions in the patient or her sexual partner^20^.

With these measurements, sexual dysfunction was studied with the exposure (pelvic floor disorders) considering predictors (e.g. pelvic floor disorders scores) as well as potential confounders (e.g. age, BMI, smoking, alcohol consumption, etc.) and effect modifiers (e.g. type of delivery, etc.).

### Definitions

*Menopause*

The time of life when a woman’s ovaries stop producing hormones and menstrual periods stop. Natural menopause usually occurs around the age of 50 years. A woman is said to be in menopause when she has not had a period for 12 months in a row^8,9^.

*Pelvic floor disorders (PFDs)*

These include urinary/fecal inconsistence, pelvic prolapse, and pelvic pain. A PFD occurs when the muscles or connective tissues of the pelvic area weaken or are injured. The most common PFDs are urinary incontinence, fecal incontinence, and pelvic organ prolapse. PFDs are more common among older women^11,15,19^.

*Sexual dysfunction*

Sexual dysfunction includes hypoactive sexual desire dysfunction, female sexual arousal dysfunction, female orgasmic dysfunction, female genital-pelvic pain dysfunction, persistent genital arousal disorder, postcoital syndrome, hypotonic orgasm, and painful orgasm^20-27^.

### Ethical considerations

This study obtained approval from the Research Ethics Committee of the province of Jaén, with the reference number SPCV-0220/0302-N-20, on 26 March 2020. Prior to beginning the questionnaire, the participants received detailed information about the study and its objectives and expressed their informed consent before participating.

### Statistical analysis

The information processing was carried out using the IBM SPSS v.28 statistical program. First, descriptive analyses were performed using absolute and relative frequencies for categorical variables, as well as means and standard deviation (SD) for quantitative variables. Subsequently, a bivariate analysis was performed using Pearson’s chi-squared test for categorical variables and analysis of variance when the independent variables were categorical and the dependent variables were quantitative (after checking the application assumptions). In this analysis, *post hoc* tests were also performed using Dunnett’s C test, which consists of a pairwise comparison based on the studentized range and is appropriate when the variances are unequal. In addition, this same analysis was performed using the non-parametric Kruskal-Wallis test due to the violation of normality. Finally, a multivariate analysis was performed using binary logistic regression to control confusion. Odds ratios (ORs) and adjusted odds ratios (AORs) were calculated with 95% confidence intervals.

## RESULTS

A total of 197 women participated. The mean age was 57.7 years (SD=8.4), with a mean BMI of 26.6 (SD=4.3); 86.3% (170) of the participants were non-smokers and 53.8% (106) drank alcohol occasionally, 50.3% (99) of the women had University level in terms of education level.

Regarding personal history, 13.6% (7) of the women had a history of gynecological disease, and 4.6% (9) had gastrointestinal disease. Regarding obstetric history, 85.8% (169) of the women had been pregnant on more than one occasion and 33.5% (66) of the women had experienced a miscarriage. Regarding the type of delivery, 78.7% (155) of the women did not undergo a caesarean section, 71.6% (141) had an episiotomy, and 37.1% (73) who suffered an episiotomy had a tear at the same time (Table 1).

**Table 1:** Sociodemographic and clinical characteristics of the study sample, a cross-sectional study, Spain 2022 (N=197)

*Characteristics*	*n (%)*
**Age** (years), mean (SD)	57.7 (8.4)
30–49.9	27 (13.7)
≥50	170 (86.3)
**BMI** (kg/m^2^)	
Normal weight <25	80 (40.6)
Overweight 25–29.9	79 (40.1)
Obese ≥30	38 (19.3)
**Civil status**	
Single	6 (3.0)
Separated	1 (0.5)
Divorced	12 (6.1)
Widowed	4 (2.0)
Common-law couple	17 (8.6)
Married	157 (79.7)
**Education level**	
Primary level, uncompleted	19 (9.6)
Primary level, completed	22 (11.2)
Secondary level	32 (16.2)
High school	25 (12.7)
University level	99 (50.3)
**Employment sector**	
Administration	17 (8.6)
Agriculture/livestock	10 (5.1)
Commerce	4 (2.9)
Industry and construction	6 (3.0)
Retired	44 (22.3)
Self-employed	25 (12.7)
Public servant	91 (46.2)
**Monthly income** (€)	
<1000	25 (12.7)
1000–1999	61 (31.0)
2000–2999	61 (31.0)
>3000	50 (25.4)
**Alcohol consumption**	
Never	43 (21.8)
Occasionally (every 2–3 weeks)	106 (53.8)
Only weekends	18 (9.1)
Frequently (one day if another)	21 (10.7)
Daily	9 (4.6)
**Smoking habit**	
No	170 (86.3)
Yes	27 (13.7)
**Pregnancy**	
0	12 (6.1)
1	16 (8.1)
≥2	169 (85.8)
**Miscarriage**	
0	131 (66.5)
1	56 (28.4)
≥2	10 (5.1)
**Vaginal birth**	
0	34 (17.3)
1	30 (15.2)
≥2	133 (67.5)
**Cesarean births**	
0	155 (78.7)
1	26 (13.2)
≥2	16 (8.1)
**Instrumental birth**	
No	116 (58.9)
Yes	81 (41.1)
**Episiotomy**	
No	56 (28.4)
Yes	141 (71.6)
**Tear**	
No	124 (62.9)
Yes	73 (37.1)
**Episiotomy and tear**	
No	96 (48.7)
Yes	101 (51.3)
**Macrosomia**	
No	149 (75.6)
Yes	48 (24.4)
**Mental health illness**	
No	194 (98.5)
Yes	3 (1.5)
**Respiratory disorder**	
No	191 (97.0)
Yes	6 (3.0)
**Gynecological disorder**	
No	190 (96.4)
Yes	7 (3.6)
**Gastrointestinal disorder**	
No	188 (95.4)
Yes	9 (4.6)

Gynecological disease: e.g. cervical, endometrial or ovarian cancer among others. Gastrointestinal disease: e.g. Crohn’s disease, gastric ulcer or irritable bowel syndrome.

Table 2 shows the distribution of the different domains that evaluate women’s sexual function (hypoactive sexual desire dysfunction, female sexual arousal dysfunction, female orgasmic dysfunction, female genital-pelvic pain dysfunction, persistent genital arousal disorder, postcoital syndrome, hypohedonic orgasm, and painful orgasm). It stands out that 51.3% (101) of the women reported having sexual dysfunction. In general terms, 79.5% (155) expressed satisfaction with their sexual health and 75.5% (148) indicated having communication with their partners in intimate relationships. However, 25.5% (50) experienced problems when initiating sexual interaction.

**Table 2:** Distribution of responses regarding women’s sexual function by category (FSM-2), a cross-sectional study, Spain, 2022 (N=197)

*Dimensions*	*Evaluation according to the Female Sexual Function (FSM-2) questionnaire*							
*Sexual response and presence of sexual dysfunction*	*Severe disorder* *n (%)*			*Moderate disorder* *n (%)*				*No disorder* *n (%)*
Sexual desire	18 (9.1)			63 (32.0)				116 (58.9)
Excitation	10 (5.1)			47 (23.9)				140 (71.1)
Lubrication	19 (9.6)			47 (23.9)				131 (66.5)
Orgasm	23 (11.7)			22 (11.2)				152 (77.2)
Problems with vaginal penetration	16 (8.1)			-				181 (91.9)
Anticipatory anxiety	4 (2.0)			6 (3.0)				187 (94.9)
**Relational aspects of sexual activity**	Absence of initiation/sex communication			Initiation/sex communication moderate				No problem with initiation/ sex communication
Sex initiation^a^	50 (25.5)			35 (17.9)				111 (56.6)
Level of sex communication^a^	26 (13.3)			22 (11.2)				148 (75.5)
**Sexual satisfaction**	Sexual dissatisfaction			Moderate				Satisfactory
Satisfaction of sexual activity^a^	11 (5.6)			19 (9.7)				166 (84.7)
General satisfaction^b^	10 (5.1)			30 (15.4)				155 (79.5)
**Descriptive categories of sexual activity**								
Reasons for sexual activity without vaginal penetration (VP)	Due to pain		Due to fear of VP	Due to lack of interest in VP	No sexual partner			Inability of partner for VP
	7 (43.8)		1 (6.3)	5 (31.3)	2 (12.5)			1 (6.3)
Frequency of sexual activity	Times per month							
	1–2	3–4		5–8		9–12		>12
	74 (38.1)	64 (33.0)		44 (22.7)		10 (5.2)		2 (1.0)
**Sexual dysfunction**								
No	96 (48.7)							
Yes*	101 (51.3)							

* Sexual dysfunction is considered present if one of the 6 first dimensions are affected.

a Missing = 1.

b Missing = 2.

Regarding symptoms, 22.9% (45) stated that they had a severe or moderate problem reaching orgasm, and 29% (57) of women stated that they had never or occasionally felt excitement in the last month. Finally, of the 16 women who had difficulties with vaginal penetration, 43.8% (7) of the women indicated that it was because they felt pain, and 31.3% (5) attributed it to a lack of interest.

Table 3 indicates the bivariate analysis between the different pelvic floor disorders and their association with sexual dysfunctions. It was observed that women who experienced urinary incontinence [49 (42.6%) vs 66 (57.4%), p=0.043] and pelvic pain [12 (25.0) vs 36 (75.0), p<0.001] were more likely to report sexual dysfunction.

**Table 3:** Bivariate and multivariate analysis of pelvic floor problems and sexual dysfunction, a cross-sectional study, Spain, 2022 (N=197)

	*Sexual dysfunction*		*Bivariate analysis*	
*Pelvic floor problems*	*No* *n (%)*	*Yes* *n (%)*	*OR (95% CI)*	*p*
**Urinary incontinence**				**0.043**
No ®	47 (57.3)	35 (42.7)	1	
Yes	49 (42.6)	66 (57.4)	**1.81 (1.02–3.21)**	
**Fecal incontinence**				0.703
No ®	88 (49.2)	91 (50.8)	1	
Yes	8 (44.4)	10 (55.6)	1.21 (0.46–3.20)	
**Pelvic pain**				**<0.001**
No ®	84 (56.4)	65 (43.6)	1	
Yes	12 (25.0)	36 (75.0)	**3.87 (1.87–8.04)**	
**Prolapse**				0.090
No ®	87 (51.2)	83 (48.8)	1	
Yes	9 (33.3)	18 (66.7)	2.10 (0.89–4.93)	
***Score impact of pelvic floor problems***	***No (N=96) Mean (SD)***	***Yes (N=101) Mean (SD)***	***Mean difference (95% CI)***	***p***
Prolapse symptoms (POPDI-6)	9.99 (11.82)	22.23 (23.31)	-12.25 (-17.49 – -7.02)	**<0.001**
Colorectal-Anal symptoms (CRADI-8)	14.84 (15.76)	22.43 (20.21)	-7.59 (-12.70 – -2.49)	**0.002**
Urinary symptoms (UDI-6)	18.71 (19.29)	32.38 (30.46)	-13.68 (-20.82 – -6.54)	**<0.001**
Pelvic function disorders Total (PFDI-20)	43.53 (40.48)	77.05 (68.41)	-33.52 (-49.24 – -17.80)	**<0.001**

® Reference categories.

The impact of pelvic floor problems was evaluated using the PFDI-20 questionnaire and its association with sexual dysfunctions. Women with sexual dysfunctions were found to have significantly higher mean scores on the POPDI-6 [9.99 (11.82) vs 22.23 (23.31), p<0.001], CRADI-8 [14.84 (15.76) vs 22.43 (20.21), p=0.002], and UDI-6 [18.71 (SD=19.29) vs 32.38 (30.46), p<0.001] subscales compared to those women without sexual dysfunction.

Table 4 below shows the results of the analysis of variance (ANOVA) and Kruskall-Wallis tests, which indicate that the mean scores on the impact of prolapse, colorectal-anal, and urinary symptoms showed statistically significant associations in most of the studied domains of sexual function. However, none of the subscales showed a statistical association with problems related to vaginal penetration. Furthermore, no statistical significance was observed between general sexual satisfaction and colorectal-anal symptoms. In addition, the mean scores, standard deviations, and different p values in the ANOVA and Kruskall-Wallis are shown, as well as the *post hoc* contrasts with the Dunnett C test.

**Table 4:** ANOVA and significance of the comparison of samples and scores on the scales used, a cross-sectional study, Spain, 2022 (N=197)

*Variable*	*Assessment of pelvic floor disorders scales*												
*Sexual response and presence of sexual dysfunction*	*n*	*POPDI-6 Mean (SD)*	*Post hoc test Dunnett C*	*Sig.*	*CRADI-8 Mean (SD)*	*Post hoc test Dunnett C*	*Sig.*	*UDI-6 Mean (SD)*	*Post hoc test Dunnett C*	*Sig.*	*PFDI-20 Mean (SD)*	*Post hoc test Dunnett C*	*Sig.*
**Sexual desire**				**<0.001** ^a^ **<0.001** ^b^			**<0.001** ^a^ **0.003** ^b^			**<0.001** ^a^ **<0.001** ^b^			**<0.001** ^a^ **<0.001** ^b^
Severe	18	44.44 (28.65)	Moderate* No*		34.03 (23.75)	Moderate No*		58.10 (35.22)	Moderate* No*		136.57 (84.56)	Moderate * No*	
Moderate	63	17.59 (19.89)	Severe* No		21.08 (19.36)	Severe No		26.39 (27.95)	Severe* No		65.06 (61.03)	Severe* No	
No	116	16.26 (12.88)	Severe* Moderate		15.08 (15.69)	Severe* Moderate		20.33 (19.90)	Severe* Moderate		46.59 (41.55)	Severe* Moderate	
**Excitation**				**<0.001** ^a^ **<0.001** ^b^			**<0.001** ^a^ **<0.001** ^b^			**<0.001** ^a^ **<0.001** ^b^			**<0.001** ^a^ **<0.001** ^b^
Severe	10	24.58 (28.29)	Moderate No		18.13 (22.57)	Moderate No		37.50 (37.68)	Moderate No		80.21 (85.79)	Moderate No	
Moderate	47	31.29 (25.62)	Severe No*		29.12 (22.30)	Severe No*		41.76 (33.36)	Severe No*		102.17 (75.64)	Severe No*	
No	140	10.63 (12.53)	Severe Moderate*		15.29 (15.42)	Severe Moderate*		19.49 (19.79)	Severe Moderate*		44.41 (40.87)	Severe Moderate*	
**Lubrication**				**<0.001** ^a^ **<0.001** ^b^			**<0.001** ^a^ **<0.001** ^b^			**<0.001** ^a^ **<0.001** ^b^			**<0.001** ^a^ **<0.001** ^b^
Severe	19	17.76 (22.82)	Moderate No		17.43 (18.87)	Moderate No		30.48 (30.05)	Moderate No		65.68 (67.02)	Moderate No	
Moderate	47	30.23 (25.81)	Severe No*		29.06 (21.61)	Severe No*		41.49 (33.85)	Severe No*		100.78 (76.11)	Severe No*	
No	131	11.04 (13.06)	Severe Moderate*		15.22 (15.86)	Severe Moderate*		19.37 (19.80)	Severe Moderate*		44.63 (41.69)	Severe Moderate*	
**Orgasm**				**<0.001** ^a^ **<0.001** ^b^			**<0.001** ^a^ **0.004** ^b^			**<0.001** ^a^ **<0.001** ^b^			**<0.001** ^a^ **<0.001** ^b^
Severe	23	34.06 (29.80)	Moderate No*		27.99 (22.25)	Moderate No*		48.56 (34.17)	Moderate No*		110.59 (81.87)	Moderate No*	
Moderate	22	30.68 (25.15)	Severe No*		29.97 (24.96)	Severe No*		47.35 (37.01)	Severe No*		108.00 (83.22)	Severe No*	
No	152	11.49 (13.33)	Severe* Moderate*		15.70 (15.68)	Severe* Moderate*		19.13 (18.91)	Severe* Moderate*		45.33 (40.54)	Severe* Moderate*	
**Problems with vaginal penetration**				0.128^a^ 0.189^b^			0.228^a^ 0.276^b^			0.142^a^ 0.137^b^			0.122^a^ 0.138^b^
Severe	NE		NC			NC			NC			NC	
Moderate	11	25.00 (25.34)	NC		25.28 (22.98)	NC		37.12 (30.30)	NC		87.41 (71.90)	NC	
No	186	15.75 (19.13)	NC		18.35 (18.23)	NC		25.04 (26.16)	NC		58.14 (57.84)	NC	
**Anticipatory anxiety**				**<0.001**^a^ **0.005**^b^			**0.016** ^a^ **0.034** ^b^			**<0.001**^a^ **0.006**^b^			**<0.001^a^ 0.004** ^b^
Severe	4	38.54 (30.31)	Moderate No		30.47 (30.98)	Moderate No		61.46 (24.38)	Moderate No		130.47 (83.12)	Moderate No	
Moderate	6	43.06 (23.07)	Severe No		37.50 (16.87)	Severe No		50.00 (34.86)	Severe No		130.56 (65.06)	Severe No	
No	187	14.93 (18.40)	Severe Moderate		17.88 (17.98)	Severe Moderate		24.18 (25.44)	Severe Moderate		56.99 (56.12)	Severe Moderate	
**Relational aspects of sexual activity**													
**Sex initiation**				**<0.001** ^a^ **<0.001** ^b^			**<0.001** ^a^ **0.005** ^b^			**<0.001** ^a^ **<0.001** ^b^			**<0.001** ^a^ **<0.001** ^b^
Absence	50	29.42 (27.26)	Moderate* No problem*		28.00 (23.11)	Moderate No problem*		43.25 (34.43)	Moderate* No problem*		100.67 (80.98)	Moderate* No problem*	
Moderate	35	14.40 (15.99)	Absence* No problem		17.14 (18.23)	Absence No problem		25.12 (26.34)	Absence* No problem		56.67 (52.28)	Absence* No problem	
No problem	111	10.85 (12.72)	Absence* Moderate		15.01 (14.72)	Absence* Moderate		18.13 (17.50)	Absence* Moderate		42.98 (37.32)	Absence* Moderate	
**Level of sex communication**				**<0.001** ^a^ **<0.001** ^b^			**<0.001** ^a^ **<0.001** ^b^			**<0.001** ^a^ **<0.001** ^b^			**<0.001** ^a^ **<0.001** ^b^
Absence	26	42.63 (27.19)	Moderate* No problem*		41.23 (23.03)	Moderate* No problem*		62.98 (34.92)	Moderate* No problem*		146.83 (80.94)	Moderate* No problem*	
Moderate	22	15.34 (20.14)	Absence* No problem		13.49 (16.88)	Absence* No problem		23.48 (26.87)	Absence* No problem		52.32 (58.44)	Absence* No problem	
No problem	148	11.71 (13.49)	Absence* Moderate		15.52 (14.91)	Absence* Moderate		19.59 (18.39)	Absence* Moderate		46.83 (38.73)	Absence* Moderate	
**Sexual satisfaction**													
**Satisfaction of sexual activity**				**<0.001** ^a^ **<0.001** ^b^			**<0.001**^a^ **0.005**^b^			**<0.001** ^a^ **<0.001** ^b^			**<0.001** ^a^ **<0.001** ^b^
**Dissatisfaction**	10	42.08 (37.45)	Moderate Satisfatory		31.25 (26.84)	Moderate Satisfatory		50.00 (37.01)	Moderate Satisfatory		123.33 (99.75)	Moderate Satisfatory	
Moderate	30	30.42 (25.19)	Dissatisfaction Sastisfactory*		29.69 (24.11)	Dissatisfaction Sastisfactory*		52.36 (35.54)	Dissatisfaction Sastisfactory*		112.47 (80.96)	Dissatisfaction Sastisfactory*	
Satisfactory	155	11.85 (13.52)	Dissatisfaction Moderate*		15.73 (15.56)	Dissatisfaction Moderate*		19.25 (18.70)	Dissatisfaction Moderate*		45.83 (40.22)	Dissatisfaction Moderate*	
**General sexual satisfaction**				**0.003** ^a^ **0.030** ^b^			0.514^a^ 0.485^b^			**0.003** ^a^ **0.010** ^b^			**0.011** ^a^ **0.052^b^**
Dissatisfaction	11	27.65 (26.89)	Moderate Satisfactory		24,15 (18.91)	Moderate Satisfactory		43.94 (26.83)	Moderate Satisfactory		95.74 (69.93)	Moderate Satisfactory	
Moderate	19	27.41 (27.33)	Dissatisfaction Satisfactory		20.72 (22.85)	Dissatisfaction Satisfactory		38.60 (35.43)	Dissatisfaction Satisfactory		86.73 (80.91)	Dissatisfaction Satisfactory	
Satisfactory	166	14.18 (17.36)	Dissatisfaction Moderate		18.70 (18.05)	Dissatisfaction Moderate		23.11 (24.55)	Dissatisfaction Moderate		55.41 (54.00)	Dissatisfaction Moderate	

* The mean difference is significant at the 0.005 level.

a p-value ANOVA test.

b p-value Kruskall-Wallis test. NC: not calculated.

Finally, a multivariate analysis (Table 5) was carried out to control for confounding and determine which factors were associated with sexual dysfunction. Specifically, pelvic floor dysfunction evaluated through the PFDI-20 scale was identified as the main risk factor, observing that women who had a higher score on this scale were more likely to present sexual dysfunction (AOR=1.01; 95% CI: 1.01–1.02).

**Table 5:** Bivariable and multivariate analysis of the factors associated with sexual dysfunction, a cross-sectional study, Spain, 2022 (N=197)

*Variable*	*Sexual dysfunction*		*Bivariate analysis*		*Multivariate analysis (adjusted regression analysis)*	
	*No* *n (%)*	*Yes* *n (%)*	*AOR* *(95% CI)*	*p*	*AOR* *(95% CI)*	*p*
**Age** (years)				0.239		0.815
30–49.9 ®	16 (59.3)	11 (40.7)	1		1	
≥50	80 (47.1)	90 (52.9)	1.64 (0.72–3.73)		1.12 (0.44–3.22)	
**BMI** (kg/m^2^)				0.227	1.02 (0.95–1.10)	0.540
**Smoking habit**				0.111		0.732
No ®	79 (46.5)	91 (53.5)	1		1	
Yes	17 (63.0)	10 (37.0)	0.51 (0.22–1.18)		0.50 (0.21–1.23)	
**Pregnancy**				0.916		0.380
0 ®	6 (50.0)	6 (50.0)	1		1	
1	7 (43.8)	9 (56.3)	1.29 (0.29–5.77)		0.39 (0.05–3.22)	
≥2	83 (49.1)	86 (50.9)	1.04 (0.32–3.34)		0.24 (0.02–2.77)	
**Vaginal birth**				0.190		0.196
0 ®	21 (61.8)	13 (38.2)	1		1	
1	12 (40.0)	18 (60.0)	2.42 (0.89–6.63)		5.48 (0.83–26.12)	
≥2	63 (47.4)	70 (52.6)	1.80 (0.83–3.88)		4.08 (0.47–35.48)	
**Cesarean birth**				0.497		0.878
0 ®	73 (47.1)	82 (52.9)	1		1	
1	13 (50.0)	13 (50.0)	0.89 (0.39–2.04)		1.22 (0.38–3.92)	
≥2	96 (48.7)	101 (51.3)	0.53 (0.19–1.54)		1.78 (0.19–16.73)	
**Instrumental birth**				0.315		0.501
No ®	60 (51.7)	56 (48.3)	1		1	
Yes	36 (44.4)	45 (55.6)	1.34 (0.76–2.37)		0.78 (0.39–1.59)	
**Episiotomy**				0.241		0.846
No ®	31 (55.4)	25 (44.6)	1		1	
Yes	65 (46.1)	76 (53.9)	1.45 (0.78–2.70)		0.90 (0.32–2.53)	
**Tear**				0.642		0.634
No ®	62 (50.0)	62 (50.0)	1		1	
Yes	34 (46.6)	39 (53.4)	1.15 (0.64–2.05)		0.84 (0.41–1.72)	
**Mental health illness**				0.089		0.999
No ®	96 (49.5)	98 (50.5)	1		1	
Yes	0 (0)	3 (100)	NC		NC	
**Respiratory disorder**				0.443		0.765
No ®	94 (49.2)	97 (50.8)	1		1	
Yes	2 (33.3)	4 (66.7)	1.94 (0.35–10.83)		0.71 (0.11–4.58)	
**Gynecological disorder**				0.650		0.845
No ®	92 (48.4)	98 (51.6)	1		1	
Yes	4 (57.1)	3 (42.9)	0.70 (0.15–3.23)		0.84 (0.14–4.99)	
**Gastrointestinal disorder**						0.279
No ®	91 (48.4)	97 (51.6)	1	0.675	1	
Yes	5 (55.6)	4 (44.4)	0.75 (0.20–2.88)		0.43 (0.10–1.90)	
**PFDI-20 questionnaire score**, Median (SD)	43.53 (40.48)	77.01 (68.41)	1.01 (1.01–1.02)	**<0.001**	1.01 (1.01–1.02)	**<0.001**

AOR: adjusted odds ratio. BMI: body mass index. PFDI-20: Pelvic Floor Distress Inventory. Values in bold are statistically significant. ®Reference categories.

## DISCUSSION

The women who experienced urinary incontinence and pelvic pain, had a higher frequency of sexual dysfunction. Specifically, pelvic floor dysfunction evaluated through the PFDI-20 scale was identified as the main risk factor, observing that menopausal women who had a higher score on this scale were more likely to present sexual dysfunction.

Sexual health in women is affected during menopause, and there are studies that, among other factors, relate it to the presence of pelvic floor dysfunctions^14,21^. Incidence reported by Verbeek and Hayward^16^ on sexual dysfunction in women with pelvic floor disorders amounts to 50–83%, with our prevalence being 51.3% of women who suffered from sexual dysfunction.

In relation to sexual function, in a study in which 93 women with urinary incontinence participated, altered items of disorders in sexual desire, orgasmic dysfunction, and sexual satisfaction were observed^22^. It is important to note that, in our results, no statistically significant alterations were observed with vaginal penetration.

Regarding fecal incontinence, Pauls et al.^23^, in a study carried out with women with this dysfunction, identified that women presented a decrease in sexual desire, sexual satisfaction, arousal, lubrication, and orgasmic capacity, but was only significant in the desired domain, whereas Visscher et al.^24^ observed statistical significance in all domains. In the case of pelvic pain, a study with 200 women, of which 100 had this dysfunction, and in line with our results, showed that the dimensions affected were the domains of sexual desire, arousal, lubrication, orgasm, and pain^25^. Furthermore, in the study by Verit and Verit with 200 women, including 100 with pelvic pain, it was observed that women who suffered from it reported worse sexual function with respect to desire, arousal, lubrication, orgasm, satisfaction, and greater frequency and severity of pain on vaginal penetration^26^.

Pelvic pain has been described as an important risk factor in sexual dysfunction, which, combined with other symptoms, can be responsible for up to 50% of sexual dysfunctions^27^. Faubion et al.^28^ determined that the impact of these symptoms on women’s sexuality can be very important. Finally, in our study, we identified that pelvic organ prolapse affects all sexual dimensions except penetration; in contrast, Tola et al.^29^ did not find that prolapse, or any other pelvic floor dysfunction, affects female sexual function.

### Strengths and limitations

The strengths of the study include the use of validated instruments previously used in the population to determine both the sexual function of women FSM-2 and the presence of pelvic floor dysfunction in the population, PFDI-20^19,20^. On the other hand, certain limitations are recognized, such as the possible influence of selection and memory biases. However, we proceed by framing questions to minimize recall errors or focusing on recent experiences. Selection bias was addressed by defining inclusion criteria, although it may not be exempt from self-selection or under-representation of certain subgroups. The questionnaire was previously piloted with language adapted to facilitate reading and understanding at all educational levels; no further changes were required after this stage. However, we acknowledge that may be present, since the sampling method was convenient. Additionally, confounding bias was avoided by including all the variables that could impact the results obtained in the multivariate analysis. With the results obtained from this study, the basis could be established to develop appropriate therapeutic and preventive strategies, considering the high prevalence of this problem and its significant impact on the sexual health of a stratum of society that is especially vulnerable and ignored in this regard, menopausal women.

## CONCLUSIONS

Pelvic floor dysfunctions in menopausal women are associated with their sexual health. Pelvic floor dysfunctions symptoms that influence sexual function are colo-rectal, urinary and prolapse. Pelvic floor disorders such as urinary incontinence and pelvic pain are those that most influence sexual function.

## Supplementary Material



## Data Availability

The data supporting this research are available from the authors on reasonable request.
